# Cyanidin-3-*O*-glucoside inhibits epithelial-to-mesenchymal transition, and migration and invasion of breast cancer cells by upregulating KLF4

**DOI:** 10.29219/fnr.v64.4240

**Published:** 2020-09-28

**Authors:** Dahu Chen, Mei Yuan, Qin Ye, Xing Wang, Jing Xu, Guangyi Shi, Zhaodi Hu

**Affiliations:** 1 School of Life Sciences, Shandong University of Technology, Zibo, China; 2 School of Life Sciences, Jiangsu Normal University, Xuzhou, China

**Keywords:** anthocyanin, cell migration, cell invasion, KLF4, FBXO32

## Abstract

**Background:**

Anthocyanins (ACNs) are capable of suppressing breast cancer growth; however, investigation on the effect and mechanism of ACNs on epithelial-to-mesenchymal transition (EMT), and cell migration and invasion in breast cancer cells is limited. A complete understanding of those properties may provide useful information on of how to use these natural compounds for cancer prevention and treatment.

**Objectives:**

The aim of this work was to investigate the role of cyanidin-3-*O*-glucoside (Cy3G), one of the most widely distributed ACNs in edible fruits, in the EMT process, and cell migration and invasion of breast cancer cells, and its underlying molecular mechanisms of how Cy3G establishes these functional roles in these cells.

**Methods:**

MDA-MB-231 and MDA-MB-468 breast cancer cells were treated with Cy3G (20 μM) for 24 h, and then the cells were used for cell migration and invasion assay. Western blotting, luciferase assay, ubiquitination assay, gene knockdown, and cycloheximide chase assay were performed to analyze the molecular mechanisms of Cy3G in suppressing EMT, and cell migration and invasion.

**Results:**

Cy3G inhibited the EMT process in these cells and significantly suppressed the migration and invasion of breast cancer cells (*P* ≤ 0.05) by upregulating Krüppel-like factor 4 (KLF4) expression at protein level. KLF4 knockdown in MDA-MB-231 cells did not reveal any change in EMT marker expression, and cell migration and invasion upon treatment with Cy3G (*P* ≥ 0.05), which strongly indicated that the effects of Cy3G were mediated by KLF4. Furthermore, we determined that Cy3G indirectly upregulated KLF4 expression by downregulating FBXO32, which is the E3 ligase of KLF4.

**Conclusion:**

Cy3G is a potential anticancer reagent as it can inhibit EMT and breast cancer cell migration and invasion by upregulating KLF4.

## Popular scientific summary

- Anthocyanins widely exist in edible plants and have anti-oxidant, anti-proliferative, and anti-inflammatory capabilities. Some of them show the prevention and inhibition effects on cancers.
- This study demonstrates that cyanidin-3-O-glucoside, one of the most widely distributed anthocyanin family members in edible fruits, can inhibit EMT, and cell migration and invasion of breast cancer cells by indirectly regulating KLF4 expression.
- Cyanidin-3-O-glucoside, a natural compound existing in edible plants, is a potential anti-metastatic reagent.

Breast cancer is the most common malignancy in women worldwide and currently ranks second among the causes of cancer-related deaths in women (1). Breast cancer begins as a local disease but can metastasize to various other organs (2). Metastasis is not only a complex process but also the major cause of cancer-related deaths. Although significant progresses have been made in treating breast cancer, metastatic dissemination is still thought of as an incurable condition and has been attributed to 500,000 deaths worldwide each year (3). Thus, there is a pressing need to identify novel molecular markers that may serve as therapeutic targets and effective reagents for controlling and treating breast cancer metastasis more effectively.

The metastatic cascade is a complicated process, in which tumor cells can survive in new organ sites that are distant from the primary tumor site by changing their genetic and epigenetic characteristics. Previous researches have indicated that epithelial-to-mesenchymal transition (EMT) is one of the major mechanisms accounting for invasiveness and metastasis of various cancers (4, 5). EMT is a process by which epithelial cells lose their characteristics, such as cell–cell adhesion and cell polarity, and acquire migratory and invasive properties. EMT is indispensable for various developmental processes, such as mesoderm formation and neural tube formation, and has also been shown to occur in the initiation of metastasis for cancer progression. Initiation of metastasis requires invasion, which is enabled by EMT, that is regulated by transcription factors (6, 7), extracellular ligands (8), and microRNAs (9–12).

Krüppel-like factor 4 (KLF4), one of the members of the KLF family, is a transcription factor that occurs in eukaryotes (13). Besides being associated with normal cell functions such as growth, differentiation, and apoptosis, KLF4 also functions as either an oncogene or a tumor suppressor, depending on the cellular context, through interaction with different target genes (14). Although the role of KLF4 in breast cancer remains controversial, KLF4 has been reported to be an EMT suppressor in breast cancer cells. The expression of KLF4 is significantly downregulated during EMT in mammary epithelial cells and in breast cancer cells (15). Moreover, through the regulation of E-cadherin (16) or Snail (17), KLF4 inhibits EMT in breast cancer cells.

Anthocyanins (ACNs) are the most plentiful flavonoids that widely exist in edible plants and are the contributors of the bright colors of many fruits, vegetables, and plants (18). It has been reported that there are more than 500 types of ACNs. Based on their chemical structure, ACNs can be divided into six categories: pelargonidin, cyanidin, delphinidin, petunidin, peonidin, and malvidin. Many reports have shown that ACNs have antioxidant (19), antiproliferative (20), and anti-inflammatory (21) capabilities. Of the many ACNs, cyanidin-3-*O*-glucoside (Cy3G) is one of the most widely distributed ACN family members in edible fruits (22). Cy3G has been reported to have tumor growth inhibitory activities in colon (23), breast (24, 25) cancers, and melanoma (26). However, investigations on the effects of Cy3G on EMT are limited; therefore, we investigated the protective effect of Cy3G in breast cancer, focusing on its effects on EMT and cell migration and invasion, and studied its underlying molecular mechanisms.

## Materials and methods

### Reagents and cells

Cy3G chloride was purchased from Sigma-Aldrich (1151935, St Louis, MO, USA). MG132 was purchased from Calbiochem (Danvers, MA, USA). Human embryonic kidney 293T (HEK293T), MDA-MB-231, and MDA-MB-468 cells were purchased from the American Type Culture Collection (Manassas, VA, USA). Cells were cultured in Dulbecco’s modified Eagle’s medium (high glucose), supplemented with 10% fetal bovine serum (FBS) and 100 U/mL of antibiotics, including penicillin and streptomycin, and were housed in humidified 5% CO_2_ incubator at 37°C. For cell treatment, the logarithmic phase cells were trypsinized and seeded at a density of about 60% confluency, cultured for 24 h, and then treated with Cy3G at a concentration of 20 μM for either 24 h or 48 h. DNA transfection was performed using X-tremeGene 9 (Roche, Indianapolis, IN, USA) according to the manufacturer’s instruction.

### Plasmids

KLF4 Short Hairpin RNA (shRNA) plasmids were purchased from GeneCopoeia (Rockville, MD, USA) (Clone 1: HSH022519-13-LVRU6GP; Clone 2: HSH022519-14-LVRU6GP; Clone 3: HSH022519-16-LVRU6GP; Clone 4: HSH022519-23-LVRU6GP; scrambled clone: CSHCTR001-1-LVRU6GP).

FBXO32 promoter reporter plasmid (HPRM39018-PG02) and FBXO32 shRNA plasmids (HSH001749-31-CU6, HSH001749-32-CU6, HSH001749-33-CU6, and HSH001749-33-CU6) were obtained from GeneCopoeia.

### Lentiviral transduction

The production of lentivirus and the infection of target cells were performed as previously described (27).

#### Quantitative PCR

RNA isolation and real-time Reverse Transcription- PCR were performed according to the manufacturer’s instructions (Invitrogen, Carisbad, CA, USA). Primer sequences for qPCR analysis are as follows: 18sRNA F, 5’-ctaccacatccaaggaagca-3’ 18sRNA R, 5’-tttttcgtcactacctccccg-3’ KLF4 F, 5’-cagcttcacctatccgatccg-3’ KLF4 R, 5’-gactccctgccatagaggagg-3’ Trcp1 F, 5’-ccagactctgcttaaaccaagaa-3’ Trcp1 R, 5’-gggcacaatcatactggaagtg-3’ Trcp2 F, 5’-aagctgattgaacgaatggtacg-3’ Trcp2 R, 5’-ccacaccgccagttagattctat-3’ FBXO32 F, 5’-gcctttgtgcctacaactgaa-3’ FBXO32 R, 5’-ctgccctttgtctgacagaat-3’ FBXO22 F, 5’-cggagcaccttcgtgttga-3’ FBXO22 R, 5’-cacacactccctccataagcg-3’ Mule F, 5’ttggaccgcttcgatggaata-3’ Mule R, 5’-tgaagttcaacacagccaagag-3’. To evaluate data reproducibility, each real-time PCR reaction was conducted in triplicate. The qRT-PCR data analysis was performed using the comparative Ct (cycle threshold) method. *18sRNA* was used as an internal control gene to normalize the amount of RNA added to the first-strand cDNA synthesis reactions. The difference between the Ct of the target gene and the Ct of the reference gene (*18sRNA*) of the same sample was calculated as dCt. The difference of dCt between Cy3G treated cells and untreated cells was calculated as ddCt. The final quantitation result is presented as the fold change of target gene expression in Cy3G-treated cells related to untreated cells normalized to *18sRNA*.

### Cell growth

Cells were first cultured for 24 h and then seeded into six independent dishes at same cell density. The dishes were divided into two groups (three for each group): one group with the Cy3G treatment (20 μM) and another one without the Cy3G treatment (control). The cells were trypsinized and counted at 24, 48, and 72 h, after Cy3G treatment for each group at each time point. The results of cell counting were obtained from a TC10 Automated Cell Counter (Bio-Rad, Hercules, CA, USA).

### Wound healing

MDA-MB-231 cells were grown on six-well plate to 100% confluency, and then the cells were scratched with a sterile pipette tip to generate a wound. The cells were cultured for another 24 h in the presence (20 μM) or absence of Cy3G. The images were recorded at the time points of 0 and 24 h.

### Migration and invasion assays

The MDA-MB-231 and MDA-MB-468 cells were cultured in the presence (20 μM) or absence of Cy3G for 24 h, and then the cells were trypsinized for the migration and invasion assay. Transwell migration and Matrigel invasion assays were performed as previously described (28). The transwell system (Corning, NY, USA) was used for assays. Briefly, the cells supplemented with serum-free medium were respectively seeded into the upper chambers coated with (for invasion) or without (for migration) Matrigel. The lower chambers contained 500 μL of culture medium plus 10% FBS. After incubation for 48 h, cells on the upper chamber side of the membrane were scraped off with cotton swabs. The migrated or invaded cells were fixed with 70% ethanol and stained with 0.5% crystal violet. The cells on the lower side of the membrane were observed under microscope, and at least five pictures were taken from each membrane randomly. The stained cells were manually counted from the pictures.

### Immunoblotting

Briefly, the cells were washed with Phosphate-Buffered Saline (PBS) and harvested with ice-cold Radioimmunoprecipitation assay (RIPA) lysis buffer supplemented with protease inhibitor. The cell lysates were centrifuged, and the supernatant were subjected to 10% Sodium Dodecyl Sulphate- Polyacrylamide Gel Electrophoresis (SDS-PAGE). Western blot analysis was performed using standard methods and detected by enhanced chemiluminescence technique. The following antibodies were used: anti-β-actin (Sigma, 610182), anti-Myc (TransGen Biotech, HT101-02, Beijing, China), anti-KLF4 (Abcam, ab151733, Cambridge, MA, USA), anti-p21 (Proteintech, 60214-1-1g, Rosemont, IL, USA), anti-FBXO32 (Proteintech, 12866-1AP), anti-E-cadherin (Santa Cruz Biotech, SC-8426, Dallas, TX, USA), anti-N-cadherin (Santa Cruz Biotech, SC-271386), and anti-viementin (Santa Cruz Biotech, SC-6260).

### Ubiquitination assay

The MDA-MB-231 cells were grown in 10-cm dishes to semi-confluency, and then transfected with HA-ubquitin expression plasmid using X-tremeGene9 (Roche) according to the manufacturer’s instructions. After 24 h of transfection, the cells were treated with Cy3G at a concentration of 20 μM for another 48 h. The cells were treated with MG132 (10 μM) for 6 h before collection. The collected cells were lysed, and the lysate was immunoprecipitated for 2 h with an anti-KLF4 antibody and then with protein G-Sepharose beads (GE Healthcare, Chicago, USA) overnight. The proteins were separated by SDS-PAGE, and then blotted with anti-HA antibody and anti-KLF4 antibody.

### Luciferase assay

The HEK 293T cells were seeded using the same density in six-well plates at 60% confluency and transfected with X-tremeGENE9 (Roche). The FBXO32 promoter firefly luciferase reporter construct (400 ng) and the pRL-SV40 Renilla luciferase construct (1 ng, for normalization) were used in co-transfection. After 8 h of transfection, the cells were treated with Cy3G (10 and 40 μM, each concentration for three wells) or no treatment (control, three wells). The cells were harvested and cell extracts were prepared after transfection for 60 h, and the luciferase activity was measured using the Dual-Luciferase Reporter Assay System (Promega, Madison, WI, USA).

### Cycloheximide chase assay

The MDA-MB-231 cells were seeded in 6-cm dishes at 60% confluency and cultured for 24 h. After a further 24 h culture in the presence of Cy3G (at a concentration of 20 μM) or in the absence of Cy3G, the cells were treated with 10 μg/mL Cycloheximide (CHX). At 0, 1, 2, and 4 h after CHX addition, the cells were harvested and washed in PBS, and then lysed on ice in RIPA buffer. The supernatant was subjected to SDS-PAGE and western blotting.

### Statistical analysis

Unless otherwise noted, each sample was assayed in triplicate (three separate cell cultures). Cell proliferation and migration/invasion assays were repeated three to four times. The *in vitro* biochemical and molecular biological experiments were repeated two or three times. Unless otherwise noted, data were presented as mean ± SEM, and the two-tailed Student’s t test was used to compare the two groups. The differences were considered statistically significant when the P values were <0.05. (*) and (**) represent *P* values less than 0.05 and 0.01, respectively.

## Results

### Cy3G does not suppress MDA-MB-231 cell growth but dramatically inhibits MDA-MB-231 and MDA-MB-468 cell migration and invasion

**Fig. 1:**
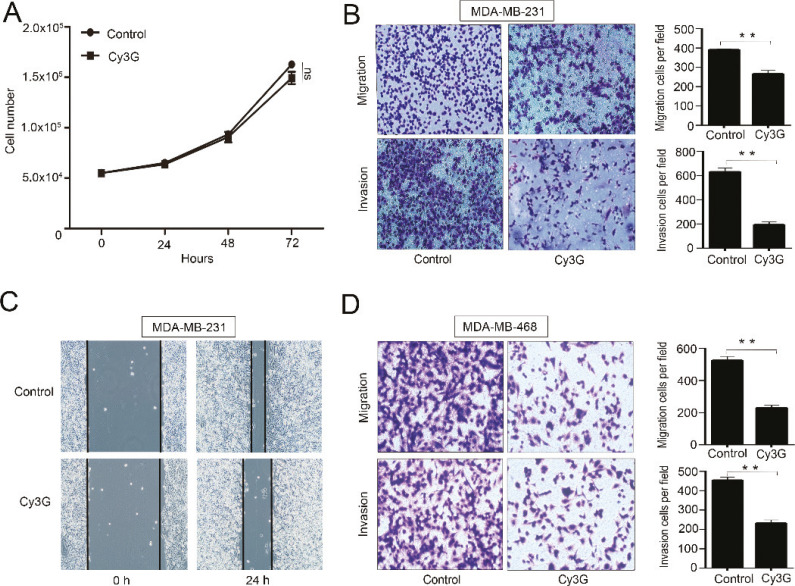
Breast cancer cell growth and migration/invasion after treatment with cyanidin-3-*O*-glucoside. (A) Growth curve of MDA-MB-231 cell in the presence or absence of Cy3G. Values are presented as mean ± SEM, *P* > 0.05. Results were from three separate cell cultures at each time point. The experiments were repeated for three or four times. (B) Representative images (magnification ×100) and statistical results of Transwell migration assays and Matrigel invasion assays of MDA-MB-231 cells in the presence or absence of Cy3G, *P* < 0.01. (C) Representative images (magnification, ×40) of the wound healing assay using MDA-MB-231 cells in the presence or absence of Cy3G. (D) Representative images (magnification, ×100) and statistical results of Transwell migration assays and Matrigel invasion assays using MDA-MB-468 cells in the presence or absence of Cy3G, *P* < 0.01. Both migration and invasion assay results were from three separate cell cultures, and the assays were repeated three or four times. Values are presented as mean ± SEM.

We initially examined the effects of Cy3G on the growth of MDA-MB-231 cells. Cy3G treatment at a concentration of 20 μM did not have any visible effects on the growth of MDA-MB-231 cells compared with the control cells (*P* = 0.1923) (Fig. 1A). Next, we studied the effects of Cy3G on cell migration. Treatment with Cy3G significantly inhibited cell migration, as determined by both wound healing experiments and Transwell migration (Fig. 1B, top panel and 1C). Because cell invasion is the first step in the initiation of cancer metastasis, we assessed the effects of Cy3G on cell invasion by Matrigel invasion assay and found that Cy3G dramatically inhibited the MDA-MB-231 cell invasion (Fig. 1B, bottom panel). To prove that the effects of Cy3G on breast cancer cell migration and invasion are not cell specific, we used another cell line MDA-MB-468 to perform the same experiments. The results indicated that Cy3G also inhibited MDA-MB-468 cell migration and invasion (Fig. 1D).

### Cy3G inhibits EMT by upregulating KLF4 expression

**Fig. 2:**
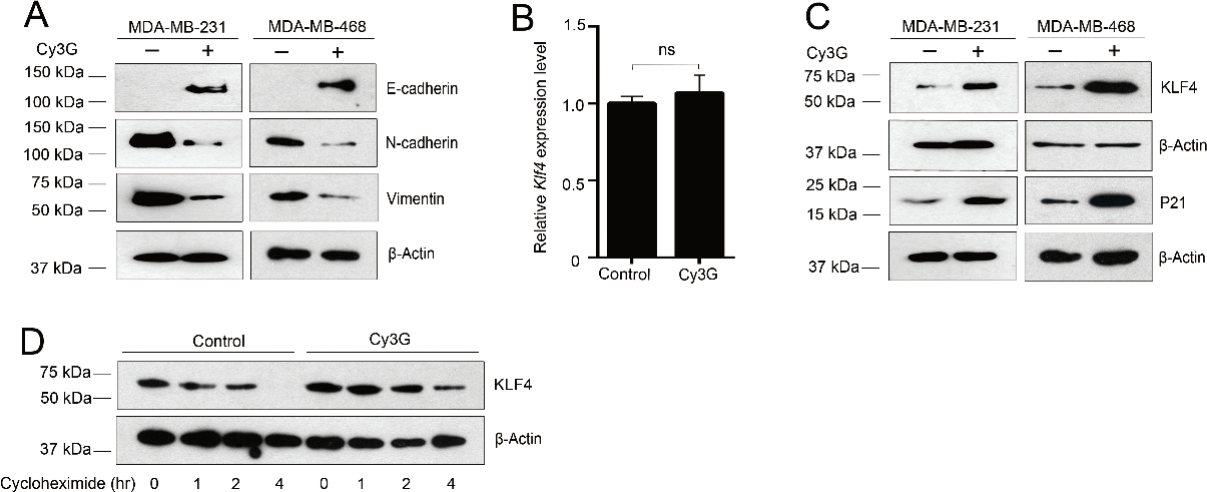
Expression of KLF4 and EMT marker genes in breast cancer cells treated with cyanidin-3-*O*-glucoside. (A) Expression of EMT marker genes in MDA-MB-231 and MDA-MB-468 cells in the presence or absence of Cy3G. (B) Expression of KLF4 at the transcriptional level in MDA-MB-231 cells in the presence or absence of Cy3G. (C) The stability of the KLF4 protein in MDA-MB-231 cells in the presence or absence of Cy3G. (D) Expression of KLF4 and P21 at the protein level in MDA-MB-231 and MDA-MB-468 cells in the presence or absence of Cy3G.

Because the initiation of metastasis requires invasion, which is enabled by EMT, we tested whether Cy3G treatment affected EMT status of the MDA-MB-231 and MDA-MB-468 cells. Although we did not detect any obvious differences in cell morphology after treatment with Cy3G in both cell lines (data not shown), we did observe changes in the expression of several EMT markers. Cy3G treatment partially restored the expression of E-cadherin and decreased the expression of the mesenchymal markers N-cadherin and vimentin in either the MDA-MB-231 cells or the MDA-MB-468 cells (Fig. 2A).

KLF4 belongs to the Krüppel-like transcription factor family that plays important roles in various fundamental biologic processes. It has been reported that KLF4 upregulates the expression of E-cadherin and inhibits EMT. We next examined whether the treatment with Cy3G affected the expression of KLF4 in MDA-MB-231 cells. The RT-qPCR results indicated that Cy3G treatment did not increase KLF4 transcription levels (Fig. 2B). However, the KLF4 protein level in the MDA-MB-231 and MDA-MB-468 cells was dramatically increased after treatment with Cy3G (Fig. 2C). To further confirm that Cy3G can upregulate KLF4, we examined the expression of P21, a typical KLF4 downstream gene. We found that the increased level of KLF4 after treatment with Cy3G did lead to an increase in P21 levels (Fig. 2C).

A cycloheximide chase assay was performed to determine whether Cy3G treatment stabilizes KLF4 protein expression levels. After treatment with CHX for 4 h, KLF4 was barely detected in the control cells. However, in Cy3G-treated cells, KLF4 remained at relatively high levels (Fig. 2D).

**Fig. 3:**
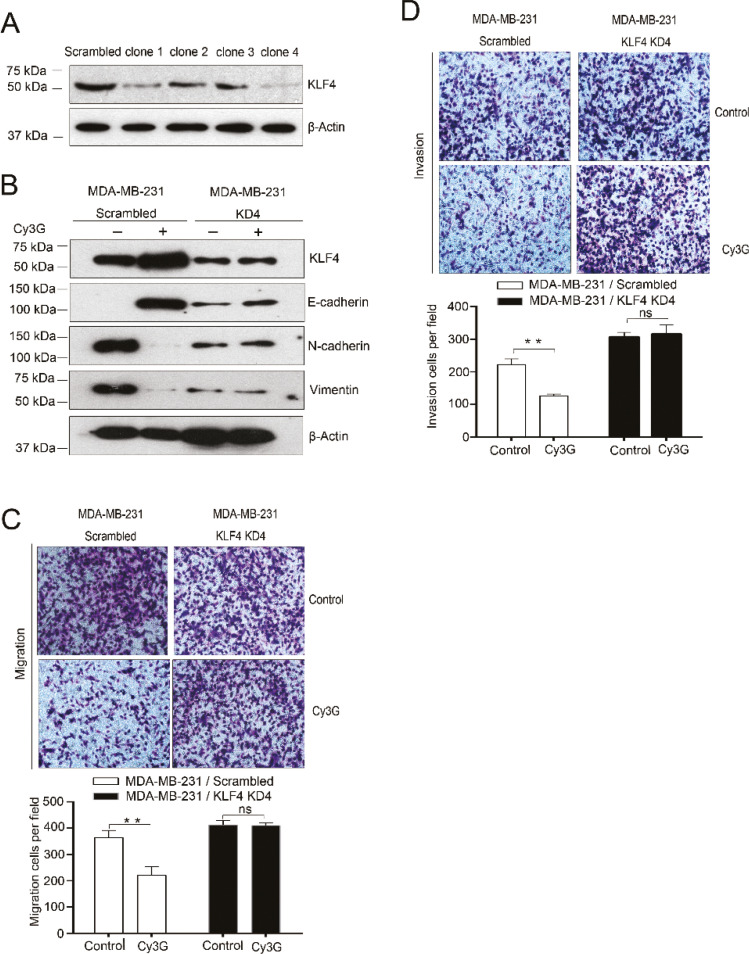
Expression of EMT marker genes and migration and invasion of KLF4-knockdown MDA-MB-231 cells after treatment with cyanidin-3-*O*-glucoside. (A) KLF4 protein expression level in 293T cells transfected either with scrambled or with KLF4 shRNAs. (B) Expression of EMT marker genes in scrambled MDA-MB-231 and in KLF4-knockdown MDA-MB-231 cells in the presence or absence of Cy3G. (C) Representative images (magnification, ×100) and statistical results of Transwell migration assays of KLF4-knockdown and scrambled MDA-MB-231 cells in the presence or absence of Cy3G, *P* > 0.05. (D) Representative images (magnification, ×100) and statistical results of Matrigel invasion assays of KLF4-knockdown and scrambled MDA-MB-231 cells in the presence or absence of Cy3G, *P* > 0.05. Both migration and invasion assay results were from three separate cell cultures, and the assays were repeated three or four times. The values are expressed as mean ± SEM.

To determine whether the changes in the expression of EMT markers were mediated by KLF4, we generated KLF4-knockdown MDA-MB-231 cell lines by shRNAs. First, we tested the KLF4 shRNA efficiency in 293T cells. Immunoblotting results showed that the highest KLF4-knockdown efficiency was achieved by shRNA clone 4 (Fig. 3A). Therefore, we established a KLF4-knockdown MDA-MB-231 cell line by transducing the shRNA into cells. In scrambled shRNA cells, Cy3G treatment leads to higher KLF4 protein expression levels and changes in the expression of EMT markers. However, in KLF4-knockdown cells, we barely observed an increase in KLF4 protein expression levels and did not observe changes in the expression of EMT markers (Fig. 3B). These results indicated that KLF4 is a crucial mediator in Cy3G treatment-induced EMT inhibition.

### Cy3G treatment does not inhibit KLF4-knockdown MDA-MB-231 cell migration and invasion

Our results thus far have indicated that Cy3G inhibits EMT by upregulating KLF4 expression. Invasion is a direct consequence of having undergone EMT and is a requirement for metastasis; therefore, we examined whether Cy3G treatment had any effects on the invasive ability of KLF4-knockdown MDA-MB-231 cells. The Matrigel invasion assay results clearly showed that Cy3G treatment did not decrease the cell invasion ability any further when KLF4 expression was suppressed to a very low level in MDA-MB-231 cells (Fig.3D, comparing top and bottom, right side).

We also performed the Transwell migration assay to analyze the cell migration upon treatment by Cy3G in KLF4-knockdown MDA-MB-231 cells. Similar to the invasion results, Cy3G treatment did not decrease cell migratory ability in KLF4-knockdown MDA-MB-231 cells any further (Fig. 3C, comparing top and bottom, right side). These data indicated that the effects of Cy3G treatment on either cell migration or invasion were dependent on KLF4.

### Cy3G increases KLF4 protein expression levels by downregulating FBXO32

**Fig. 4:**
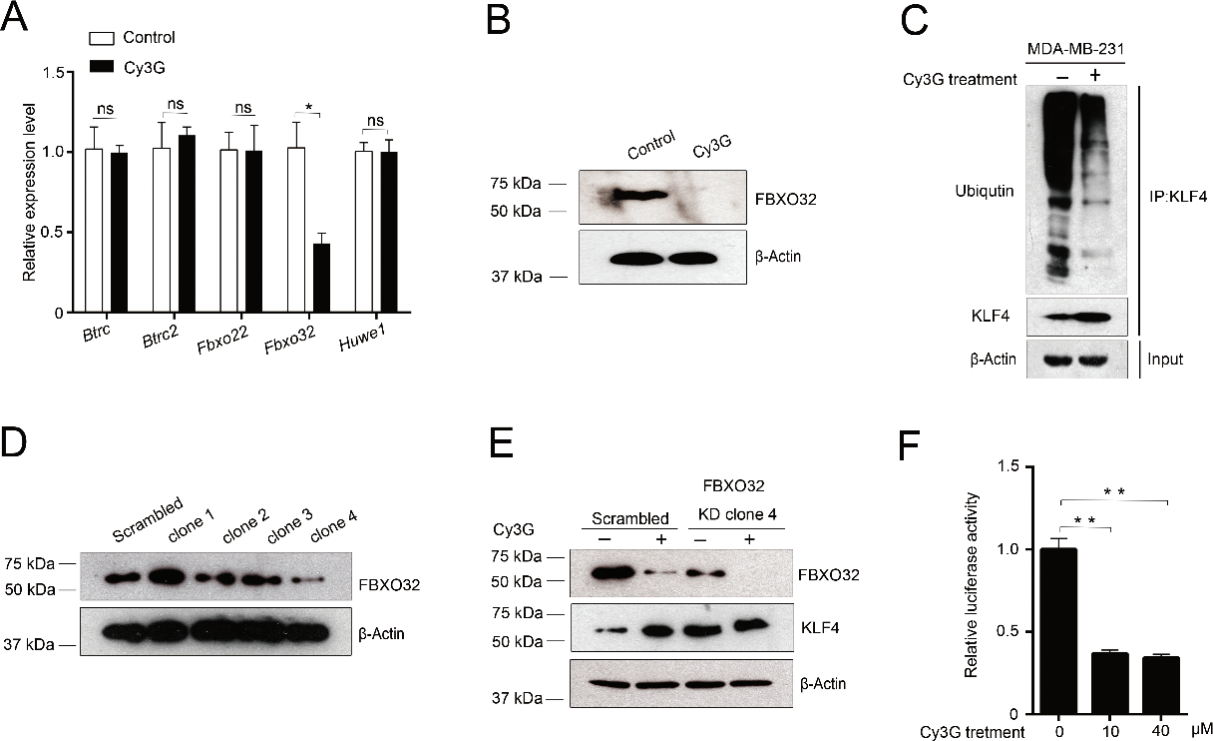
Indirect upregulation of KLF4 expression by cyanidin-3-*O*-glucoside through downregulating of FBXO32. (A) Expression of *Fbxo32* and other KLF4 E3 ligase genes at the transcription level in MDA-MB-231 cells in the presence or absence of Cy3G. (B) Expression of FBXO32 at the protein level in MDA-MB-231 cells in the presence or absence of Cy3G. (C) The ubiquitination level of KLF4 in MDA-MB-231 cells in the presence or absence of Cy3G. (D) The protein expression level of FBXO32 in MDA-MB-231 cells transfected either with scrambled or with FBXO32 shRNAs. (E) The expressions of FBXO32 and KLF4 in scrambled and FBXO32-knockdown MDA-MB-231 cells in the presence or absence of Cy3G. (F) Relative luciferase activity of FBXO32 promoter in the presence or absence of Cy3G. Results were from three separate transfections, and the assays were repeated three times. The values are presented as mean ± SEM.

Cy3G treatment increased KLF4 protein expression levels (Fig. 2C). One of the possible explanations for this phenomenon is that Cy3G stabilizes KLF4. The results of the cycloheximide chase assay confirmed that Cy3G did stabilize KLF4 (Fig. 2D). Ubiquitination/de-ubiquitination is one of the most important protein posttranslational modification mechanisms for regulating protein stability. We hypothesized that Cy3G influences KLF4 ubiquitination or de-ubiquitination. Because no investigations on KLF4 de-ubiquitination enzymes have been conducted to date, we focused on the effects of Cy3G treatment on KLF4 ubiquitination. To verify our hypothesis, we performed RT-qPCR to detect changes in the expression of several identified KLF4 E3 ligases after Cy3G treatment. Our qPCR results indicated that Cy3G treatment only decreased FBXO32 expression (Fig. 4A). Immunoblotting results also confirmed the downregulation of FBXO32 by Cy3G treatment (Fig. 4B). Due to the reduction in the expression level of FBXO32 after treatment with Cy3G, the KLF4 ubiquitination level decreased (Fig. 4C), which in turn stabilized KLF4. To clarify that the regulatory effect of Cy3G on KLF4 expression is mediated by FBXO32, we established a FBXO32-knockdown MDA-MB-231 cell line (Fig. 4D). When FBXO32 was knocked down, Cy3G treatment did not significantly increase the KLF4 protein expression levels compared to the scrambled cell line (Fig. 4E).

To further confirm the regulatory effect of Cy3G on FBXO32 expression, we performed a FBXO32 promoter reporter luciferase assay. Our results indicated that Cy3G at a concentration of 10 μM could effectively inhibit FBXO32 promoter activity (Fig. 4F). Thus, by downregulating the expression of FBXO32, which caused a reduction of the ubiquitination level of KLF4, Cy3G indirectly upregulated KLF4, which led to an inhibition of EMT and cell migration and invasion.

## Discussion

Several studies have confirmed that the activation of the EMT program promotes tumor cell invasion and metastasis. Metastasis is a significant hallmark of malignancy, and significantly influences patient prognosis, treatment plan, and quality of life. Developing specific drugs against cancer metastasis is still a huge challenge. However, by specifically targeting the cancer cells undergoing EMT, some novel drugs and therapies against metastasis are expectable. In our current study, we demonstrated that (1) Cy3G, which is widely distributed in edible fruits and vegetables, inhibits EMT and breast cancer cell migration and invasion, (2) the effects of Cy3G on EMT and breast cancer cell migration and invasion are mediated by KLF4, and (3) mechanically, Cy3G inhibits the expression of the KLF4 E3 ligase, FBXO32, leading to stabilization of KLF4 and suppression of EMT and cell migration and invasion. These findings lay a theoretical foundation and provide proof of concept for using natural food extracts to control cancer metastasis.

ACNs are natural phytochemicals that are abundantly found in many edible plants and are bioactive dietary agents. Owing to their numerous potential health benefits, including interference with several processes involved in cancer development and progression (29–31), ACNs have received considerable attention. Previous studies have positively correlated the dietary consumption of ACNs with reduced cardiovascular disease-associated mortality (32, 33) and with the inhibition of certain types of cancer (34–36). Previous findings have suggested the potential of ACNs or ACN-derived pigments for use in chemotherapy for breast cancer (37). Cy3G, one member of the ACNs family present in various vegetables and fruits, especially edible berries, has been confirmed to have antioxidant properties (38). Cy3G also has been implicated in specific beneficial health actions, including reducing age-associated oxidative stress (39), improving cognitive brain function (40) as well as antidiabetic (41), anti-inflammation (42), and anti-obesity activities (43). Cy3G exhibits anticancer properties in various *in vitro* and animal models of carcinogenesis and tumor development; however, the mechanisms of those effects established by Cy3G are still elusive.

In the present study, we first observed that MDA-MB-213 cell growth was not suppressed by the treatment of Cy3G. It seems contradictory that the expression of P21, a downstream target gene of KLF4 and an inhibitor of cell cycle progression, increased after the cells were treated with Cy3G, yet no change in cell growth was observed. One possible explanation for this is that Cy3G directly regulated FBXO32. FBXO32, an E3 ligase, may have multiple substrates, including some already identified and some unknown targets. Those substrates may exert an antagonistic role to compensate the cell cycle blocking function of P21. We then focused on the effect of Cy3G on cell migration and invasion. We found that Cy3G treatment dramatically inhibits MDA-MB-231 and MDA-MB-468 cells migration and invasion. Adams et al. reported that the phytochemicals from blueberries inhibit MDA-MB-231 metastatic capability (44). Our results further confirm that some components of ACNs have the potential ability to inhibit cell migration, invasion, and metastasis either *in vitro* or *in vivo*. Because we did not observe that Cy3G influences cell growth, the inhibition of tumor migration and invasion established by Cy3G in this study was unlikely due to the decreased cell proliferation.

EMT is the first in the cascade of steps that are involved in tumor invasion and metastasis. The original malignant epithelial cells lose intercellular junctions and lumen-basement polarity and thus develop the capacity to migrate, cross basement membranes, and invade blood vessels. Our results showed that Cy3G could partially recover the expression of the epithelial marker E-cadherin and decrease the expression of mesenchymal markers N-cadherin and vimentin. EMT has attracted a lot of attention as a potential mechanism for tumor cell metastasis. EMT is regulated by transcription factors, extracellular ligands, and miRNAs. KLF4 is one of the transcription factors that have been found to regulate EMT. Although the function of KLF4 in breast cancer remains controversial, it is well established that KLF4 inhibits EMT in breast cancer cells. Here, we demonstrated that Cy3G increased KLF4 expression, which leads to the induction of E-cadherin and reduction of N-cadherin and vimentin in MDA-MB-231 and MDA-MB-468 cells. In KLF4 knockdown cells, Cy3G lost the ability to inhibit cell migration and invasion and did not change EMT marker expression any further. To the best of our knowledge, this might be the first study to report that KLF4 can be upregulated by Cy3G, which leads to the inhibition of EMT and cell migration and invasion.

Protein posttranslational modification is an important mechanism for regulating gene expression, and ubiquitination is one of the major forms of protein posttranslational modification. Because Cy3G only regulates KLF4 expression at the protein level, ubiquitination may be involved in this process. Therefore, we performed RT-qPCR assay to determine whether the expression of the known E3 ligase for KLF4 can be regulated by Cy3G. Among the tested E3 ligases, only FBXO32 expression was decreased by Cy3G treatment. FBXO3 is an identified E3 ligase for KLF4. FBXO3 physically interacts with the N-terminus of KLF4 via its C-terminus and directly targets KLF4 for ubiquitination and degradation (45). *In vitro* ubiquitination assay results indicated that Cy3G treatment significantly decreased KLF4 ubiquitination status, which explained the observation that KLF4 expression increase only occurred at the protein level after treatment with Cy3G. However, this data did not provide evidence to prove that the upregulation effect of Cy3G on KLF4 is through FBXO3. To clarify the role of FBXO3 in this process, we established FBXO3-knockdown cell line. When FBXO32 was knocked down to low level, Cy3G treatment only limitedly decreased FBXO32 expression and thus did not significantly increase KLF4 protein expression levels compared to the scrambled cell line. These data demonstrated that when treatment with Cy3G, the KLF4 expression changed with the expression of FBXO32, its E3 ligase. In terms of the function of FBXO32, Zhou et al. (45) recently found that FBXO32 functions as a tumor suppressor in breast cancer. However, in our case, FBXO32 acted as a promoter of EMT and cell migration/invasion. Some reports have shown that EMT and cell migration/invasion are not always positively correlated with tumor development (46, 47). In addition, besides KLF4, FBXO32 can target some other unidentified substrates that are important in EMT, cell migration/invasion, and tumor development to establish its dual functions.

In sum, our data show that Cy3G inhibits EMT, and cell migration and invasion by upregulating KLF4 through the transcriptional suppression of FBXO32 expression. Our study has revealed a novel function of Cy3G in EMT, and in migration and invasion of breast cancer cells, and provides an insight into the mechanism whereby a natural compound that exists in edible plants imparts anti-metastatic effects on breast cancer.

## Authors’ contribution

D.C. conceived and designed research; Y.M. performed most of the experiments. D.C. and Y.M. analyzed data; Q.Y., X.W., J.X, G.S., and Z.H. provided scientific supports; D.C. wrote the paper. All authors read and approved the final manuscript.

## References

[1] Matamala N, Vargas MT, Gonzalez-Campora R, Minambres R, Arias JI, Menendez P, et al. Tumor microRNA expression profiling identifies circulating microRNAs for early breast cancer detection. Clin Chem 2015; 61(8): 1098–106. doi: 10.1373/clinchem.2015.238691 26056355

[2] Weigelt B, Peterse JL, van’t Veer LJ Breast cancer metastasis: markers and models. Nat Rev Cancer 2005; 5(8): 591–602. doi: 10.1038/nrc1670 16056258

[3] Gallo S, Sangiolo D, Carnevale Schianca F, Aglietta M, Montemurro F Treating breast cancer with cell-based approaches: an overview. Expert Opin Biol Ther 2017; 17(10): 1255–64. doi: 10.1080/14712598.2017.1356816 28728493

[4] Chaffer CL, San Juan BP, Lim E, Weinberg RA EMT, cell plasticity and metastasis. Cancer Metastasis Rev 2016; 35(4): 645–54. doi: 10.1007/s10555-016-9648-7 27878502

[5] Lambert AW, Pattabiraman DR, Weinberg RA Emerging biological principles of metastasis. Cell 2017; 168(4): 670–91. doi: 10.1016/j.cell.2016.11.037 28187288 PMC5308465

[6] Thiery JP Epithelial-mesenchymal transitions in tumour progression. Nat Rev Cancer 2002; 2(6): 442–54. doi: 10.1038/nrc822 12189386

[7] Yang J, Weinberg RA Epithelial-mesenchymal transition: at the crossroads of development and tumor. Dev Cell 2008; 14(6): 818–29. doi: 10.1016/j.devcel.2008.05.009 18539112

[8] Scheel C, Onder T, Karnoub A, Weinberg RA Adaptation versus selection: the origins of metastatic behavior. Cancer Res 2007; 67(24): 11476–9. doi: 10.1158/0008-5472.CAN-07-1653 18089773

[9] Gregory PA, Bert AG, Paterson EL, Barry SC, Tsykin A, Farshid G, et al. The miR-200 family and miR-205 regulate epithelial to mesenchymal transition by targeting ZEB1 and SIP1. Nat Cell Biol 2008; 10(5): 593–601. doi: 10.1038/ncb1722 18376396

[10] Wellner U, Schubert J, Burk UC, Schmalhofer O, Zhu F, Sonntag A, et al. The EMT-activator ZEB1 promotes tumorigenicity by repressing stemness-inhibiting microRNAs. Nat Cell Biol 2009; 11(12): 1487–95. doi: 10.1038/ncb1998 19935649

[11] Shimono Y, Zabala M, Cho RW, Lobo N, Dalerba P, Qian D, et al. Downregulation of miRNA-200c links breast cancer stem cells with normal stem cells. Cell 2009; 138(3): 592–603. doi: 10.1016/j.cell.2009.07.011 19665978 PMC2731699

[12] Ma L, Young J, Prabhala H, Pan E, Mestdagh P, Muth D, et al. miR-9, a MYC/MYCN-activated microRNA, regulates E-cadherin and cancer metastasis. Nat Cell Biol 2010; 12(3): 247–56. doi: 10.1038/ncb2024 20173740 PMC2845545

[13] Song X, Xing YM, Wu W, Cheng GH, Xiao F, Jin G, et al. Expression of Kruppel-like factor 4 in breast cancer tissues and its effects on the proliferation of breast cancer MDA-MB-231 cells. Exp Ther Med 2017; 13(5): 2463–67. doi: 10.3892/etm.2017.4262 28565864 PMC5443194

[14] Rowland BD, Peeper DS KLF4, p21 and context-dependent opposing forces in cancer. Nat Rev Cancer 2006; 6(1): 11–23. doi: 10.1038/nrc1780 16372018

[15] Tiwari N, Meyer-Schaller N, Arnold P, Antoniadis H, Pachkov M, van Nimwegen E, et al. Klf4 is a transcriptional regulator of genes critical for EMT, including Jnk1 (Mapk8). PLoS One 2013; 8(2): e57329. doi: 10.1371/journal.pone.0057329 23451207 PMC3581489

[16] Yori JL, Johnson E, Zhou G, Jain MK, Keri RA Kruppel-like factor 4 inhibits epithelial-to-mesenchymal transition through regulation of E-cadherin expression. J Biol Chem 2010; 285(22): 16854–63. doi: 10.1074/jbc.M110.114546 20356845 PMC2878056

[17] Yori JL, Seachrist DD, Johnson E, Lozada KL, Abdul-Karim FW, Chodosh LA, et al. Kruppel-like factor 4 inhibits tumorigenic progression and metastasis in a mouse. Neoplasia 2011; 13(7): 601–10. doi: 10.1593/neo.11260 21750654 PMC3132846

[18] Wu X, Beecher GR, Holden JM, Haytowitz DB, Gebhardt SE, Prior RL Concentrations of anthocyanins in common foods in the United States and estimation of normal consumption. J Agric Food Chem 2006; 54(11): 4069–75. doi: 10.1021/jf060300l 16719536

[19] Higgins JA, Zainol M, Brown K, Jones GD Anthocyans as tertiary chemopreventive agents in bladder cancer: anti-oxidant mechanisms and interaction with mitomycin C. Mutagenesis 2014; 29(4): 227–35. doi: 10.1093/mutage/geu009. 24743948

[20] Chen PN, Chu SC, Chiou HL, Chiang CL, Yang SF, Hsieh YS Cyanidin 3-glucoside and peonidin 3-glucoside inhibit tumor cell growth and induce apoptosis in vitro and suppress tumor growth in vivo. Nutr Cancer 2005; 53(2): 232–43. doi: 10.1207/s15327914nc5302_12 16573384

[21] Zhang H, Yousef H, Renaud J, Liu R, Yang C, Sun Y, et al. Bioaccessibility, bioavailability and anti-inflammatory effects of anthocyanins from purple root vegetable using mono and coculture cell models. Mol Nutr Food Res 2017; 61(10). doi: 10.1002/mnfr.201600928 28691370

[22] Wu X, Prior RL Systematic identification and characterization of anthocyanins by HPLC-ESI-MS/MS. In common foods in the United States: fruits and berries. J Agric Food Chem 2005; 53(7): 2589–99. doi: 10.1021/jf048068b 15796599

[23] Serra D, Paixao J, Nunes C, Dinis TCP, Almeida LM Cyanidin-3-glucoside suppresses cytokine-induced inflammatory response in human intestinal cells: comparison with 5aminosaliccylic acid. PLoS One 2013; 8(9): e73001. doi: 10.1371/journal.pone.0073001 24039842 PMC3765207

[24] Xu M, Bower KA, Wang S, Frank JA, Chen G, Ding M, et al. Cyanidin-3-glucoside inhibits ethanol-induced invasion of breast cancer cells overexpressing ErbB2. Mol Cancer 2010; 9: 285. doi: 10.1186/1476-4598-9-285 21034468 PMC2984473

[25] Wang L, Li H, Yang S, Ma W, Liu M, Guo S, et al. Cyanidin-3-o-glucoside directly binds to ERalpha36 and inhibits EGFR-positive triple negative breast cancer. Oncotarget 2016; 7(42): 68864–82. doi: 10.18632/oncotarget.12025 27655695 PMC5356596

[26] Liu M, Du Y, Li H, Wang L, Ponikwicka-Tyszko D, Lebiedzinska W, et al. Cyanidin-3-o-glucoside pharmacologically inhibits tumorigenesis via estrogen receptor β in melanoma mice. Front Oncol 2019; 9: 1110. doi: 10.3389/fonc.2019.01110 31696058 PMC6817467

[27] Stewart SA, Dykxhoorn DM, Palliser D, Mizuno H, Yu EY, An DS, et al. Lentivirus-delivered stable gene silencing by RNAi in primary cells. RNA 2003; 9(4): 493–501. doi: 10.1261/rna.2192803 12649500 PMC1370415

[28] Ma L, Teruya-Feldstein J, Weinberg RA Tumour invasion and metastasis initiated by microRNA-10b in breast cancer. Nature 2007; 449(7163): 682–8. doi: 10.1038/nature06174 17898713

[29] Khoo HE, Azlan A, Tang ST, Lim SM Anthocyanidins and anthocyanins: colored pigments as food, pharmaceutical ingredients, and the potential health benefits. Food Nutr Res 2017; 61(1): 1361779. doi: 10.1080/16546628.2017.1361779 28970777 PMC5613902

[30] Kristo AS, Klimis-Zacas D, Sikalidis AK Protective role of dietary berries in cancer. Antioxidants (Basel) 2016; 5(4): E37. doi: 10.3390/antiox5040037 27775562 PMC5187535

[31] Grimes KL, Stuart CM, McCarthy JJ, Kaur B, Cantu EJ, Forester SC Enhancing the cancer cell growth inhibitory effects of table grape anthocyanins. J Food Sci 2018; 83(9): 2369–74. doi: 10.1111/1750-3841.14294 30070707

[32] Kimble R, Keane KM, Lodge JK, Howatson G Dietary intake of anthocyanins and risk of cardiovascular disease: a systematic review and meta-analysis of prospective cohort studies. Crit Rev Food Sci Nutr 2019; 59(18): 3032–3043. doi: 10.1080/10408398.2018.1509835 30277799

[33] Liobikas J, Skemiene K, Trumbeckaite S, Borutaite V Anthocyanins in cardioprotection: a path through mitochondria. Pharmacol Res 2016; 113(Pt. B): 808–15. doi: 10.1016/j.phrs.2016.03.036 27038533

[34] Wang Y, Lin J, Tian J, Si X, Jiao X, Zhang W, et al. Blueberry Malvidin-3-galactoside suppresses hepatocellular carcinoma by regulating apoptosis, proliferation, and metastasis pathways in vivo and in vitro. J Agric Food Chem 2019; 67(2): 625–36. doi: 10.1021/acs.jafc.8b06209 30586992

[35] Han B, Peng X, Cheng D, Zhu Y, Du J, Li J, et al. Delphinidin suppresses breast carcinogenesis through the HOTAIR/microRNA-34a axis. Cancer Sci 2019; 110(10): 3089–97. doi: 10.1111/cas.14133 31325197 PMC6778627

[36] Diaconeasa Z, Ayvaz H, Ruginǎ D, Leopold L, Stǎnilǎ A, Socaciu C, et al. Melanoma inhibition by anthocyanins is associated with the reduction of oxidative stress biomarkers and changes in mitochondrial membrane potential. Plant Foods Hum Nutr 2017; 72(4): 404–10. doi: 10.1007/s11130-017-0638-x 29129015

[37] Liu W, Xu J, Liu Y, Yu X, Tang X, Wang Z, et al. Anthocyanins potentiate the activity of trastuzumab in human epidermal growth factor receptor 2-positive breast cancer cells in vitro and in vivo. Mol Med Rep 2014; 10(4): 1921–26. doi: 10.3892/mmr.2014.2414 25070704

[38] Sousa A, Araújo P, Azevedo J, Cruz L, Fernandes I, Mateus N, et al. Antioxidant and antiproliferative properties of 3deoxyanthocyanidins. Food Chem 2016; 192: 142–8. doi: 10.1016/j.foodchem.2015.06.108 26304331

[39] Pacheco SM, Soares MSP, Gutierres JM, Gerzson MFB, Carvalho FB, Azambuja JH, et al. Anthocyanins as a potential pharmacological agent to manage memory deficit, oxidative stress and alterations in ion pump activity induced by experimental sporadic dementia of Alzheimer’s type. J Nutr Biochem 2018; 56: 193–204. doi: 10.1016/j.jnutbio.2018.02.014 29587242

[40] Min J, Yu SW, Baek SH, Nair KM, Bae ON, Bhatt A, et al. Neuroprotective effect of cyanidin-3-O-glucoside anthocyanin in mice with focal cerebral ischemia. Neurosci Lett 2011; 500(3): 157–61. doi: 10.1016/j.neulet.2011.05.048 21651957

[41] Qin Y, Zhai Q, Li Y, Cao M, Xu Y, Zhao K, et al. Cyanidin-3-O-glucoside ameliorates diabetic nephropathy through regulation of glutathione pool. Biomed Pharmacother 2018; 103: 1223–30. doi: 10.1016/j.biopha.2018.04.137 29864902

[42] Ma B, Wu Y, Chen B, Yao Y, Wang Y, Bai H, et al. Cyanidin-3-O-β-glucoside attenuates allergic airway inflammation by modulating the IL-4Rα-STAT6 signaling pathway in a murine asthma model. Int Immunopharmacol 2019; 69: 1–10. doi: 10.1016/j.intimp.2019.01.008 30660871

[43] Bhaswant M, Fanning K, Netzel M, Mathai ML, Panchal SK, Brown L Cyanidin 3-glucoside improves diet-induced metabolic syndrome in rats. Pharmacol Res 2015; 102: 208–217. doi: 10.1016/j.phrs.2015.10.006 26477387

[44] Adams LS, Kanaya N, Phung S, Liu Z, Chen S Whole blueberry powder modulates the growth and metastasis of MDA-MB-231 triple negative breast tumor in nude mice. J Nutr 2011; 141(10): 1805–12. doi: 10.3945/jn.111.140178 21880954 PMC3174855

[45] Zhou H, Liu Y, Zhu R, Ding F, Wan Y, Li Y, et al. FBXO32 suppresses breast cancer tumorigenesis through targeting KLF4 to proteasomal degradation. Oncogene 2017; 36(23): 3312–21. doi: 10.1038/onc.2016.479 28068319 PMC5926769

[46] Celià-Terrassa T, Meca-Cortés O, Mateo F, Martínez de Paz A, Rubio N, Arnal-Estapé A, et al. Epithelial-mesenchymal transition can suppress major attributes of human epithelial tumor-initiating cells. J Clin Invest 2012; 122(5): 1849–68. doi: 10.1172/JCI59218 22505459 PMC3366719

[47] Chen D, Sun Y, Yuan Y, Han Z, Zhang P, Zhang J, et al. miR-100 induces epithelial-mesenchymal transition but suppresses tumorigenesis, migration and invasion. PLoS Genet 2014; 10(2): e1004177. doi: 10.1371/journal.pgen.1004177 24586203 PMC3937226

